# Modulation of Early‐Life Calf Gut Microbiota by Bacteriocin‐Producing *Lactiplantibacillus pentosus* AA86 and *Pediococcus pentosaceus* AA94

**DOI:** 10.1002/mbo3.70438

**Published:** 2026-10-14

**Authors:** Adewale A. Adetoye, Ayorinde O. Afolayan, Isaac O. Olatoye, Adedayo O. Faneye, Funmilola A. Ayeni

**Affiliations:** ^1^ Department of Pharmaceutical Microbiology and Biotechnology, Faculty of Pharmacy Lead City University Ibadan Oyo State Nigeria; ^2^ Leibniz Institute DSMZ – German Collection of Microorganisms and Cell Cultures Braunschweig Germany; ^3^ Department of Veterinary Public Health and Preventive Medicine, Faculty of Veterinary Medicine University of Ibadan Oyo State Nigeria; ^4^ Department of Virology, College of Medicine University of Ibadan Oyo State Nigeria; ^5^ Department of Environmental and Occupational Health, School of Public Health Indiana University, Bloomington Indiana USA

**Keywords:** bacteriocin, calves, lactic acid bacteria, microbiota, probiotics

## Abstract

Early‐life gut microbiota plays a critical role in livestock health and productivity. Probiotic interventions, particularly those employing bacteriocin‐producing lactic acid bacteria (LAB), may contribute to modulation of microbial development during this critical period. This study evaluated *Lactiplantibacillus pentosus* AA86 (formerly *Lactobacillus pentosus*) and *Pediococcus pentosaceus* AA94 for genomic safety, bacteriocin potential, and their effects on calf gut microbiota. Whole‐genome sequencing assessed antimicrobial resistance genes, virulence determinants, plasmid‐associated elements, and bacteriocin‐encoding genes. Both strains lacked detectable antimicrobial resistance genes, virulence determinants, and plasmid‐related elements, while harboring pediocin‐encoding genes consistent with potential antimicrobial functionality. In a 30‐day feeding trial, eight calves (50 ± 10 kg) were assigned to a control group (sterile skimmed milk, *n* = 2) or a probiotic group (milk supplemented with both strains at ~8.6 × 10^10^ CFU/day, *n* = 6). Gut microbiota was evaluated at baseline, mid‐, and post‐intervention using 16S rRNA gene (V3/V4) sequencing and QIIME2. Alpha diversity and overall community structure did not differ significantly between groups. However, supplementation was associated with selective temporal compositional shifts, particularly in Firmicutes and Bacteroidetes. At the genus level, *Lactobacillus* showed significant temporal changes within treatment group, although the treatment‐by‐time interaction was not significant. *L. pentosus* AA86 and *P. pentosaceus* AA94 exhibited favorable genomic safety profiles and bacteriocin‐associated antimicrobial potential. Their supplementation was associated with selective temporal modulation of the developing calf gut microbiota without substantial alteration of overall community structure. These findings provide a foundation for larger and longer‐term studies incorporating strain‐resolved, functional, metabolomic, and host health analyses.

## Graphical abstract

Early‐life supplementation with bacteriocin‐producing LAB selectively modulated calf gut microbiota development. Overall microbial diversity remained unchanged, while LAB‐fed calves showed treatment‐associated shifts in Firmicutes and Bacteroidetes. These findings suggest selective ecosystem‐level modulation of the developing calf gut microbiota without broad distruption of the community diversity.

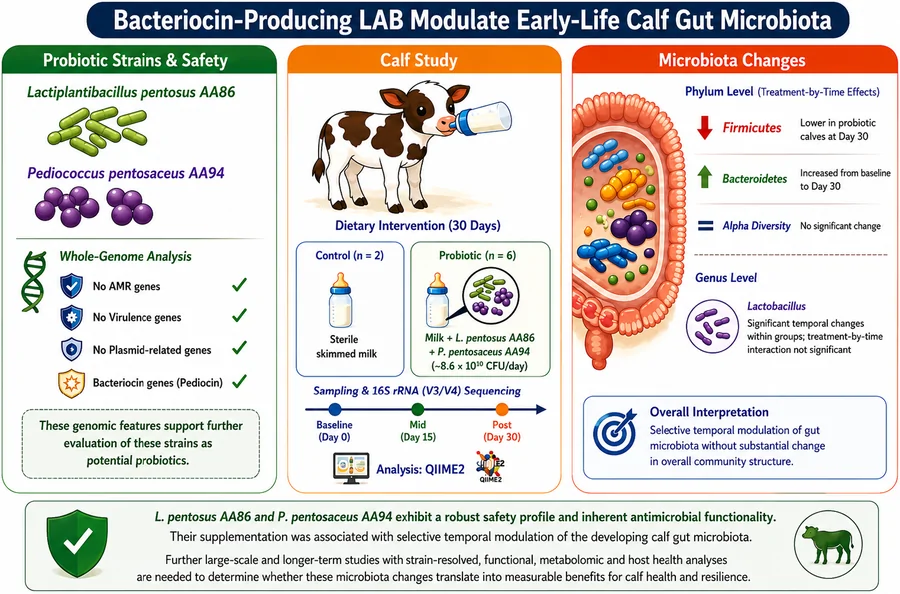

## 1 Introduction

There has been an unprecedented increase in demand for animal‐source protein over recent decades with continual projected increase, primarily due to increasing global population, urbanization and income expansion, particularly in low‐ and middle‐income countries (Henchion et al. 2017; FAO, 2018; OECD & FAO, 2024). The coping strategies for optimum productivity in modern livestock management involve growth promotion and disease prevention; these have encouraged the widespread incorporation of antibiotics as growth promoters in various livestock feed (Low et al. 2021; Omonijo et al. 2025; Zheng et al. 2025). The growth‐promoting effects of antibiotics are largely attributed to their ability to suppress intestinal pathogenic bacteria, thereby reducing sub‐clinical infections, lowering immune activation, and improving nutrient utilization and metabolic efficiency (Low et al. 2021; Miyakawa et al. 2024; Rasool et al. 2025). The routine incorporation of sub‐therapeutic doses of antibiotics into animal feed for the purpose of growth promotion has been identified as a major driver of the emergence and dissemination of antimicrobial resistance determinants among pathogenic bacteria and commensal microbiota of animal origin (Adeniyi et al. 2015; Odey et al. 2023). Other downsides include dysbiosis and persistence of antibiotic residues in animal products with far‐reaching health implications (Van Boeckel et al. 2015).

A balanced gut microflora constitutes an effective pathogen colonization barrier, produces beneficial metabolic substrate, and stimulates the immune system (Mansilla et al. 2022; Kouhounde et al. 2022). Early development of the gastrointestinal tract microflora plays an essential role in building the immune system and long‐term health of the host. Recent reports suggest that microbial colonization begins in‐utero contrary to the initial “sterile‐fetus dogma” view held for many decades (Perez‐Muñoz et al. 2017; Guzman et al. 2020; Russell et al. 2023; Bautista and López‐Cortés 2025). Neonatal calves though exposed to microorganisms from the dam's vaginal and perineum during parturition, are born with a non‐functional and undeveloped rumen (Welch et al. 2022). The microbial population of the gut begins to be established by microbes from the breast milk, skin during suckling and the oral cavity (Gomez et al. 2019). It has been established that early postnatal period is a critical window for manipulation of the gut microflora to optimize immunity in newborns (Pantazi et al. 2023).

Probiotics are increasingly recognized as effective alternatives to in‐feed antibiotics, promoting growth performance through modulation of gut microbial homeostasis, enhancement of intestinal barrier integrity, regulation of tight junction expression, stimulation of mucosal immunity, and reshaping of microbial metabolic activity, thereby reducing pathogen colonization and improving gut functionality during critical developmental windows (Li et al. 2023; Gangaiah et al. 2024; Zamojska et al. 2021; Omonijo et al. 2025; Chowdhury et al. 2025). An important feature driving these effects is the ability of specific strains to produce bacteriocins—ribosomally synthesized antimicrobial peptides that selectively inhibit competing and pathogenic microorganisms, thereby promoting competitive exclusion and stabilizing the gut microbial ecosystem (Reuben and Torres 2024; Fitsum et al. 2025). Bacteriocin production by lactic acid bacteria represents a particularly potent and ecologically relevant mechanism, as it enables selective suppression of undesirable taxa without inducing the broad microbiota disruption commonly associated with conventional antibiotics (Anjana and Tiwari 2022). Consequently, probiotic supplementation has been shown to restructure fecal microbiota composition and diversity, favoring enrichment of beneficial taxa while suppressing opportunistic pathogens, changes that correlate with improved nutrient utilization, growth performance, and immunocompetence (Wang et al. 2023; Rodríguez‐Garza et al. 2025). In calves, at the pre‐ruminant stage, probiotics are generally targeted at the lower intestine, where it is thought to stabilize the gut microbial community, reduce the risk of bacterial infection and improve the animal's general well‐being (Uyeno et al. 2015).

Despite growing interest in probiotic supplementation for livestock, the ecological interactions between administered probiotic strains and the resident gut microbiota, particularly regarding community resilience, functional redundancy, and long‐term stability remain incompletely understood and represent an important focus of current microbiome research (Moya and Ferrer 2016; Vonaesch et al. 2025). Relatively few studies have examined the effects of targeted probiotic interventions on the intestinal microbiota of calves using high‐resolution genomic approaches. This gap is particularly relevant considering the increasing global demand for animal protein and the need to improve feed efficiency, growth performance, and overall animal health in a sustainable manner. These pressures have been further intensified by regulatory restrictions on the use of in‐feed antimicrobials in livestock production, following bans implemented in the European Union and several other regions, which have necessitated the identification of safe and effective alternatives to antibiotic growth promoter (Millet and Maertens 2011; Zheng et al. 2025).

Bacteriocin production and probiotic functions are inherently strain‐specific. Therefore, careful selection of candidate strains, supported by comprehensive safety evaluations, including accurate taxonomic identification, assessment of virulence and pathogenicity, screening for antibiotic resistance determinants, and confirmation by whole‐genome sequencing remains essential for their use as probiotics in livestock (Sanders et al. 2010; Tenea, 2021). In the present study, whole‐genome sequencing was employed to evaluate the safety profile and bacteriocinogenic potential of two candidate probiotic strains, *L. pentosus* AA86 and *P. pentosaceus* AA94, previously isolated from cattle feces. Additionally, the study investigated the effect of dietary supplementation with a combined formulation of both strains on the biodiversity and taxonomic structure of the calf gut microbiota over a 30‐day intervention period.

## 2 Materials and Methods

### 2.1 Bacterial Strains

*Lactiplantibacillus pentosus* AA86 and *Pediococcus pentosaceus* AA94 were previously isolated from cattle feces on the University of Ibadan Teaching and Research Farm and extensively characterized for their probiotic properties (Adetoye et al. 2018). The cultures were preserved at ‐80°C in de Mann Rogosa Sharpe (MRS) broth containing 20% glycerol. Both strains were grown in MRS agar (Oxoid, Basingstoke, UK) at 37°C for 48 h prior to DNA extraction.

### 2.2 Genomic DNA Extraction and Genome Sequencing

Genomic DNA was extracted from the two strains according to the manufacturer's protocol using the *AccuPrep* DNA Extraction kit (Bioneer, South Korea). The DNA was checked for purity by a NanoDrop 2000 Spectrophotometer (Thermo Fisher Scientific, USA) and quantified using a Qubit 3.0 Fluorometer (Life Technologies, USA). For generation of short‐read sequencing, an Illumina NextFLEX library was sequenced on a NextSeq. 500 instrument, with 2×150 bp read length at Cornnel Genomics (NY).

### 2.3 Genome Assembly, Speciation, and Annotation

Raw sequence reads were uploaded onto Pathogenwatch website (https://pathogen.watch/) for quality control, genome assembly, species identification, and antimicrobial resistance screening. The assembled reads were screened for bacteriocin genes using the publicly available BAGEL tool (v4; http://bagel4.molgenrug.nl/) (van Heel et al. 2018). https://academic.oup.com/nar/article/46/W1/W278/5000017?login=false).

### 2.4 Ethical Statement

All animal handling procedures were reviewed and approved by the Animal Care and Use Research Ethics Committee (ACUREC) of the University of Ibadan with the approval number UI‐ACUEC/17/0011 and were conducted in accordance with national and international guidelines for animal care.

### 2.5 Preparation of Lactic Acid Bacteria Feeding Suspension

A modified method of Casey et al. (2007) was used to prepare the LAB suspension; *L*. *pentosus* AA86 and *P. pentosaceus* AA94 cells, grown separately in MRS broth, were harvested by centrifugation. The resulting cell pellets of both strains were resuspended daily in 10 mL of 10% reconstituted skimmed milk and used for the feeding trial. The colony‐forming units (CFU/ml) of each suspension batch were determined on MRS agar to ensure consistency in bacterial counts throughout the experiment.

### 2.6 Calves Feed Trial

All the calves (Eight, (weight= 50 ± 10 kg)) available at the dairy section of UI Teaching and Research Farm (UI‐T& RF) were recruited for the experiment between July and August 2018. All eight calves available in the facility were used for this study. Each calf was ear‐tagged for identification and transferred to the pathogen challenge facility of the research farm. The calves were penned individually to avoid cross‐contamination, and the control calves were kept away from the probiotic‐treated group. All calves had unrestricted access to water and their regular daily feed ration, and no antibiotic feed additive or antibiotics were administered throughout the feeding trial. The calves were randomly assigned to two treatment groups: the mixed LAB suspension group (*n* = 6), which received a combination of *L. pentosus* AA86 and *P. pentosaceus* AA94 at an approximate dose of 8.3 × 10^10^ CFU/mL daily via oral gavage, and the control group (*n* = 2), which received 10 mL of sterile reconstituted skimmed milk daily. For 30 days, the test group received 10 mL of mixed LAB‐skimmed milk suspension, while the control group received 10 mL of sterile skimmed milk.

### 2.7 Collection of Fecal Samples and Sequencing

Feces were collected directly from the rectum of each numbered, tagged calves (at three time points: before, midpoint [day 15] and on day 30 of the feeding trial). The genome DNA was extracted with the QIAamp DNA Stool Mini Extraction Kit (Qiagen, Germany) according to the manufacturer's protocol. The V3–V4 region of the 16S rRNA gene was amplified with the 341 F and 805 R primers with Illumina adaptor overhang sequences as previously described (Candela et al. 2016). The library was prepared and subsequently sequenced on the Illumina MiSeq platform using a 2 × 300 bp paired‐end protocol, according to the manufacturer's instructions (Candela et al. 2016).

### 2.8 Bioinformatics and Statistics

After sequencing, raw reads were processed using QIIME2 version 2019.7 (Bolyen et al. 2018). Demultiplexed reads were denoised with Deblur (Bokulich et al. 2013; Amir et al. 2017). A rooted phylogenetic tree was generated using the align‐to‐tree‐mafft‐fasttree (Price et al. 2010) pipeline from the q2‐phylogeny plugin. Representative sequences were classified by a pre‐trained Naïve Bayes classifier using the Greengenes database (v13_8, (McDonald et al. 2012) specific for the V3/V4 region (515 F/806 R primers) of the 16S rRNA gene.

Using a sampling depth of 1000, core metrics for alpha diversity (Shannon, observed features, faith's phylogeny, Pielou's evenness) and beta diversity (weighted and unweighted UniFrac distances (Lozupone and Knight 2005), Bray‐Curtis distances, Jaccard distances) were calculated. The alpha diversity data were imported into R (v3.5.3) as QIIME 2 artifacts using the *read_qza* function of the qiime2R package (v0.99.4) and were visualized with added statistics using the ggplot2 package (v3.3.3) and the ggpubr package (v0.4.0; implements the Wilcoxon test for pairwise comparisons). Taxonomy data were imported into R by preparing a phyloseq object using phyloseq (v1.26.1) (McMurdie and Holmes 2013) and were visualised using ggplot2. Potential functional pathways were predicted using the picrust2 plugin available in QIIME2 (https://github.com/gavinmdouglas/q2-picrust2) (Langille et al. 2013; Douglas et al. 2019).

Taxonomic read counts were analyzed using generalized linear mixed‐effects models implemented in the glmmTMB R package (v1.1.14). Counts were modeled using a negative binomial distribution (nbinom2) with a log link, with the log‐transformed total reads per sample included as an offset to account for differences in sequencing depth. Treatment (LAB vs. control), sampling time (Initial, Middle, and Final), and their interaction were included as fixed effects, while calf identity was included as a random intercept to account for repeated measurements from the same animals. The Treatment × Time interaction was used to determine whether temporal changes in taxon abundance differed between LAB‐treated and control calves. Estimated marginal means and post‐hoc pairwise comparisons were obtained using the emmeans R package (v2.0.4). P‐values for the primary Treatment × Time tests across taxa were adjusted using the Benjamini–Hochberg false discovery rate (FDR) correction, with FDR‐adjusted *p* < 0.05 considered statistically significant. Principal Coordinate analysis (PCoA) plots of beta diversity metric distances were visualized in R using the ggplot2 package. Differences in beta diversity between intervention types were calculated using adonis2. Differences in alpha diversity were calculated using the geom_pwc function in ggpubr (v0.6.2).

## 3 Results

### 3.1 Whole Genomic Analysis of Potential Probiotic Strains

The genomic analysis identified *L. pentosus* AA86 and *P. pentosaceus* AA94 as the respective strains isolated from cattle feces. Detailed characteristics of each genome assembly are presented in Table 1. *L. pentosus* AA86 exhibited a genome length of 3,665,302 base pairs, with 112 contigs, while *P. pentosaceus* AA94 had a genome length of 1,843,469 base pairs, assembled into 71 contigs. It is noteworthy that both genomes did not contain antimicrobial resistance genes, virulence factors, or plasmid‐related genes, except for the Rep28 plasmid replicon (with 97.097% identity) carried by *L. pentosus*. However, the presence of pediocin, a bacteriocin with broad‐spectrum antimicrobial activity against pathogens, was detected in both isolates (Figures 1 and 2).

**Table 1:** Basic characteristics of genome assemblies of *Lactiplantibacillus pentosus* A86 and *Pediococcus pentosaceus* AA94.

Bacterial strain	Genome length	No. of contigs	Smallest contig	Largest contig	Average contig length	N50	GC%
*Lactiplantibacillus pentosus* AA86	3,665,302	112	112	262,935	32,725	91565	46.2
*Pediococcus pentosaceus* AA‐94	1,843,469	71	147	145,112	25964	55464	37.1

**Figure 1:**
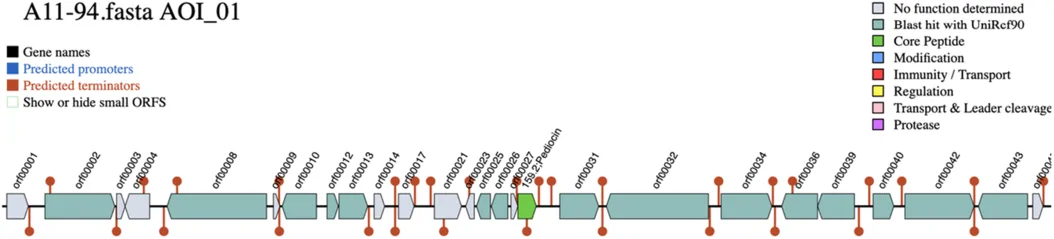
Gene map of *Pediococcus pentosaceus* AA94 showing the presence of Pediocin (Bright green), a bacteriocin.

**Figure 2:**
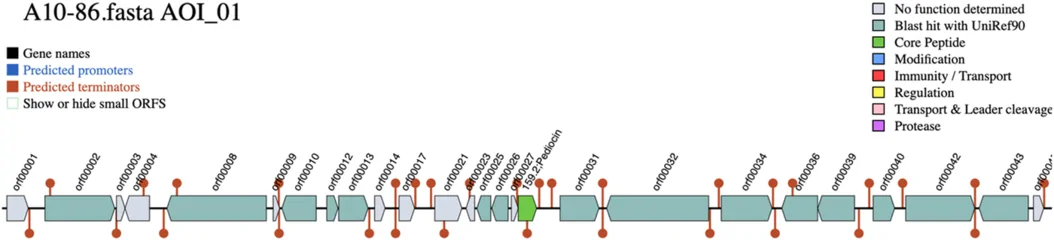
Gene map of *Lactiplantibacillus pentosus* AA86 showing the presence of Pediocin (Bright Green), a bacteriocin.

### 3.2 Effect of Probiotic Intervention on Calves' Gut Microbiome

The alpha and beta diversity analyses (adonis2; *p* = 0.6) showed no statistically significant differences among intervention stages or between intervention and control groups, indicating that overall microbial diversity remained comparable throughout the study period (Figure 3). Nevertheless, a gradual increase in diversity was observed across intervention stages relative to baseline in LAB‐fed calves, whereas diversity patterns in the control calves group were less consistent over time. Visual inspection of diversity distributions further suggested a trend toward progressive stabilization of microbial diversity in LAB‐fed calves, particularly from mid to final sampling, in contrast to the more variable patterns observed in controls.

**Figure 3:**
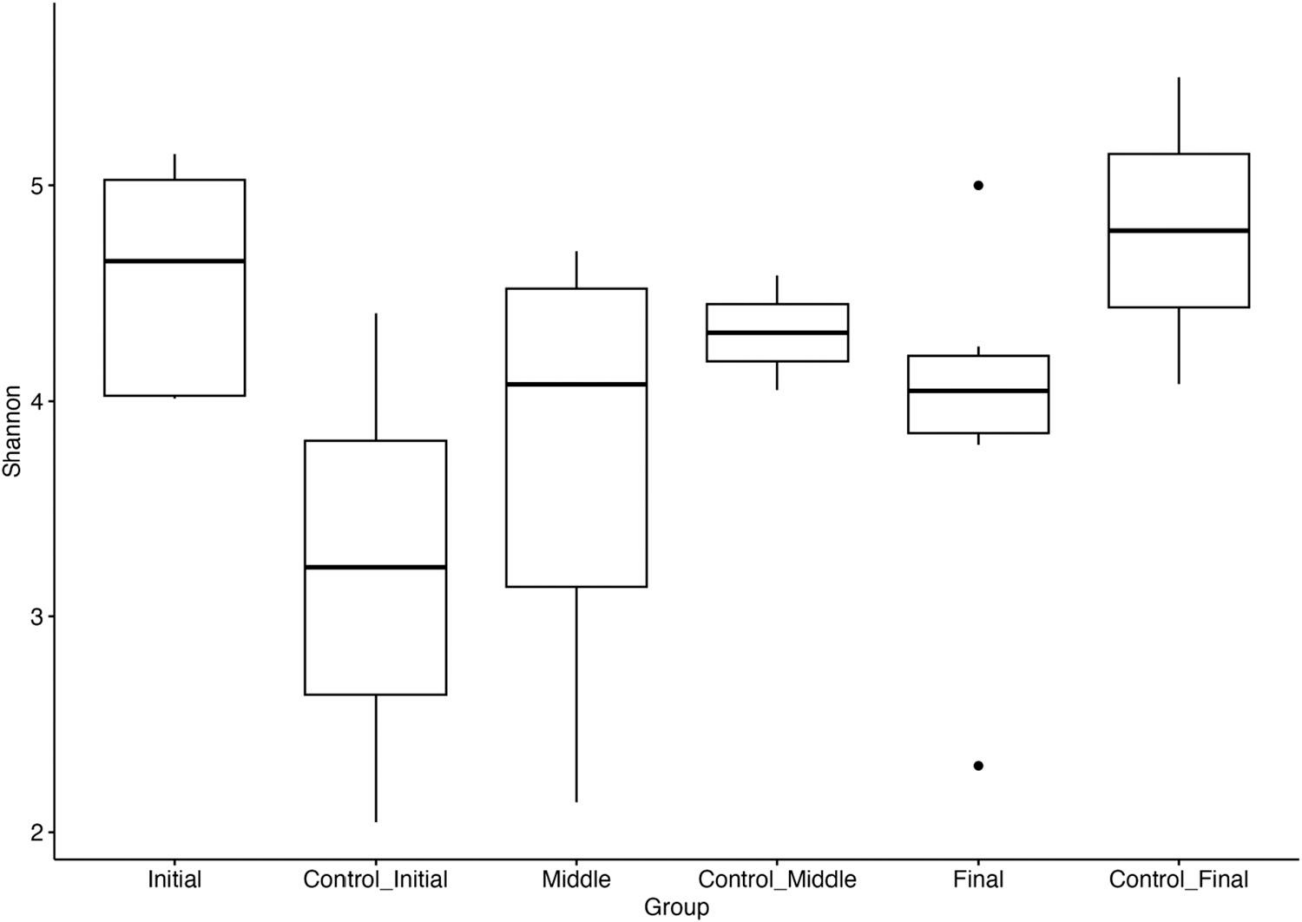
Compositional alpha diversity (Shannon) of the gut microbiota of 8 calves before, during, and after dietary intervention with lactic acid bacteria. Negative control groups were indicated as Control_Initial, Control_Middle, and Control_Final. Boxplots include data points, a box with median and interquartile ranges, and *p*‐values as determined using the Wilcoxon test. Boxplots show no significant difference in alpha diversity.

At the phylum level, Firmicutes showed a significant treatment‐by‐time interaction between the Initial and Final sampling points (FDR‐adjusted *p* = 0.048). Firmicutes abundance did not differ significantly between groups at baseline (Initial; *p* = 0.464). Among control calves, no significant changes were observed between Initial and Middle (*p* = 0.539) or Initial and Final (*p* = 0.209). The Initial‐to‐Middle change also did not differ significantly between treatments (*p* = 0.541). At the Final sampling point, estimated marginal means showed significantly lower Firmicutes abundance in LAB‐treated calves than in control calves (FDR‐adjusted *p* = 0.031; Figure 4a–b).

**Figure 4:**
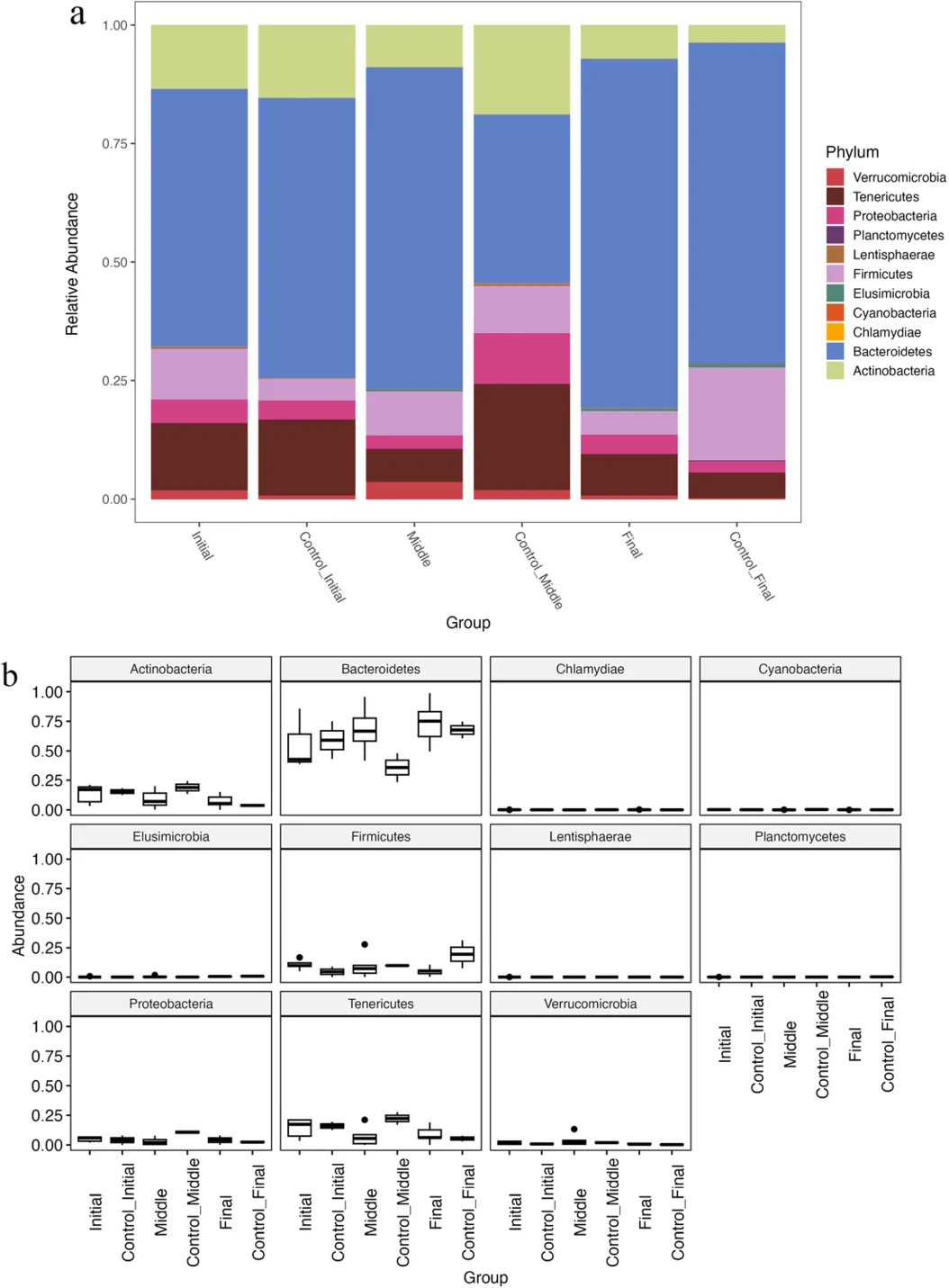
(a) Taxonomic profile of the gut microbiota at the phylum level in samples retrieved from calves under dietary intervention. Negative controls are indicated with the “Control” prefix. (b): Comparison of the relative abundance at the phylum level in samples retrieved from calves under dietary intervention. Negative controls are indicated with the “Control” prefix. Boxplots include data points, a box with median and interquartile ranges, and *p*‐values as determined using the Wilcoxon test. Boxplots show no significant difference in the relative abundance across the intervention groups.

Bacteroidetes also showed treatment‐dependent temporal changes (Figure 4a–b). Baseline abundance did not differ significantly between LAB‐treated and control calves (*p* = 0.99). Among control calves, Bacteroidetes abundance did not change significantly from Initial to Middle (*p* = 0.25) or Initial to Final (*p* = 0.40). In contrast, Bacteroidetes abundance increased significantly from Initial to Final in LAB‐treated calves (FDR‐adjusted *p* = 0.01). The Initial‐to‐Middle trajectory differed significantly between treatments (FDR‐adjusted *p* = 0.041), whereas the Initial‐to‐Final trajectory did not (*p* = 0.58). Estimated marginal means further showed significantly higher Bacteroidetes abundance in LAB‐treated calves than in controls at the Middle intervention (FDR‐adjusted *p* = 0.0018), but not at the Final intervention point (*p* = 0.400).

No significant treatment‐by‐time interaction was observed for Actinobacteria (*p* = 0.586), indicating that its temporal trajectory did not differ between LAB‐treated and control calves. Post‐hoc comparisons revealed no significant temporal changes within the LAB group, while a significant difference was observed between the Initial and Middle sampling points among control calves. Similarly, Proteobacteria showed no significant treatment‐by‐time interaction (*p* = 0.596). Neither the Initial‐to‐Middle (*p* = 0.196) nor Initial‐to‐Final (*p* = 0.174) treatment‐dependent changes were significant. Post‐hoc comparisons revealed no significant temporal differences within either treatment group. Tenericutes also showed no significant treatment‐by‐time interaction (*p* = 0.568). The treatment‐dependent changes from Initial to Middle (*p* = 0.159) and Initial to Final (*p* = 0.959) were not significant, and post‐hoc comparisons revealed no significant temporal differences within either treatment group.

At the genus level, no significant treatment‐by‐time interactions were observed (Figure 5a–b). Nevertheless, post‐hoc comparisons identified significant temporal differences within control calves. *Bacteroides*, *Parabacteroides*, *Mycoplasma*, *Shigella*, and *Staphylococcus* showed significantly lower abundance at the Final compared with the Middle sampling point, whereas *Helicobacter* abundance was significantly higher at the Final than at the Middle sampling point. In control calves, *Lactobacillus* abundance decreased significantly from Initial to Final (FDR‐adjusted *p* = 0.014) and from Middle to Final (FDR‐adjusted *p* = 0.0003). While LAB‐treated calves showed a significant decrease from Initial to Middle (FDR‐adjusted *p* = 0.0306) but no significant difference between Initial and Final.

**Figure 5:**
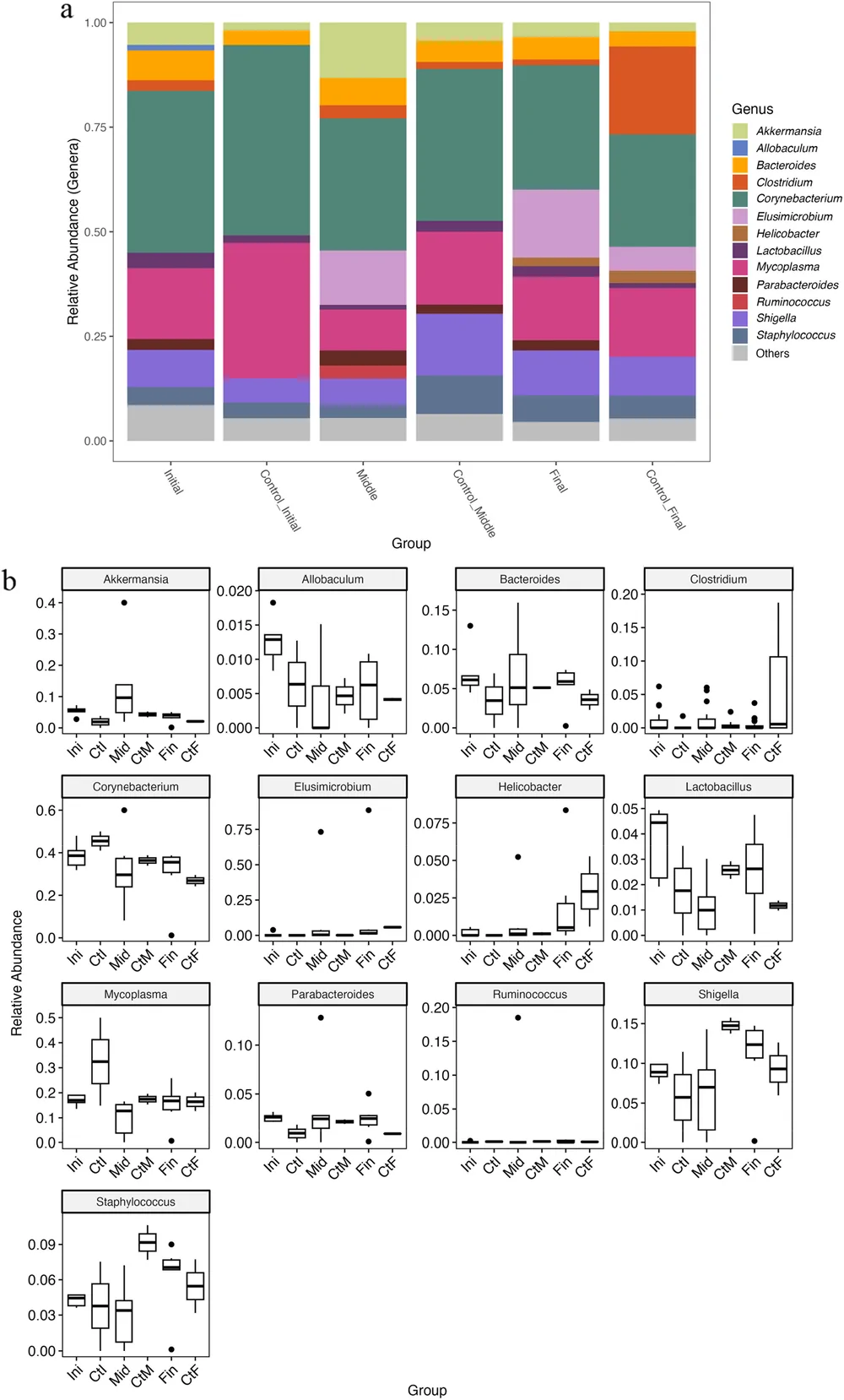
(a) Taxonomic profile of the gut microbiota at the genus level in samples retrieved from calves under dietary intervention. Negative controls are indicated with the “Control” prefix. (b) Comparison of the relative abundance at the genus level in samples retrieved from calves under dietary intervention. The abbreviation Ini, CtI, Mid, CtM, Fin, and CtF refers to Initial, Control_Initial, Middle, Control_Middle, Final, and Control_Final, respectively. Boxplots include data points, a box with median and interquartile ranges, and *p*‐values as determined using the Wilcoxon test. Boxplots show no significant difference in the relative abundance across the intervention groups.

## 4 Discussion

The selection of *Lactiplantibacillus pentosus* AA86 and *Pediococcus pentosaceus* AA94 for this intervention study was informed by their origin from cattle feces and previous characterization, which reported tolerance to acidic pH and bile, absence of hemolytic activity, organic acid production, and antimicrobial activity (Adetoye et al. 2018). Whole‐genome analysis further indicated the absence of antimicrobial resistance genes, virulence factors and plasmid‐associated elements; these characteristics provided a microbiological basis for evaluating the strains as potential probiotics in calves. In addition to safety‐related genomic features, whole‐genome analysis revealed the presence of genes encoding pediocin in both isolates, indicating their potential to produce bacteriocins with antimicrobial activity. Pediocin is a well‐characterized class II bacteriocin with documented inhibitory effects against a broad range of bacterial taxa, including several Gram‐positive pathogens and, under certain conditions, Gram‐negative organisms (Khorshidian et al. 2021; Todorov et al. 2021; Todorov et al. 2022). The detection of pediocin‐encoding genes in *L. pentosus* AA86 and *P. pentosaceus* AA94 therefore positioned these strains as promising probiotic candidates with potential antimicrobial functionality prior to their evaluation *in vivo*. This genomic profile aligns with the long‐standing application of *Lactobacillus* and *Pediococcus* species in food fermentation and preservation, where they have demonstrated safety and functional efficacy (Todorov et al. 2021; Chaichana et al. 2025).

The present intervention study demonstrates that supplementation with *Lactiplantibacillus pentosus* AA86 and *Pediococcus pentosaceus* AA94 was associated with selective changes in the gut microbial development in calves rather than broad restructuring of the bacterial community. Using 16S metagenomic sequencing, we captured community‐wide taxonomic structure across initial, mid‐intervention, and post‐intervention stages. Evidence of treatment‐associated effects was most apparent at the phylum level, particularly for Firmicutes and Bacteroidetes, whereas alpha diversity measured by the Shannon index was not significantly affected and no significant treatment‐by‐time interactions were detected at the genus level.

Shannon diversity did not differ significantly between treatment groups or across sampling stages. This finding is not unusual, as it aligns with previous studies suggesting that LAB‐based probiotics often do not markedly alter overall microbial diversity when compared with controls (De Wolfe et al. 2018; Rodenes‐Gavidia et al. 2023; Lee et al. 2025; Bajzert et al. 2025). This observation is also consistent with evidence that probiotic supplementation does not necessarily alter alpha diversity, even when changes in particular microbial taxa occur (Sanders 2016; Kristensen et al. 2016). A recent systematic review and meta‐analysis of randomized trials found no significant overall effect of probiotics on Shannon diversity, while earlier systematic reviews have equally reported minimal effects of probiotic supplementation on alpha diversity (Éliás et al. 2026). The overall pattern is consistent with the dynamic nature of the calf gut microbiota during early life, where increasing microbial diversity is not expected to occur abruptly; rather, it reflects a gradual ecological assembly process shaped by diet, host development, and microbial succession (Amin and Seifert 2020; Arshad et al. 2021; Wu et al. 2023). From this perspective, LAB supplementation did not appear to disrupt overall resident community structure during a critical early‐life window (Konieczka et al. 2023; Idowu et al. 2025).

The phylum‐level analysis provided stronger evidence of selective microbial modulation. Firmicutes showed a significant treatment‐by‐time interaction between the initial and final sampling points, with lower estimated abundance in LAB‐treated calves than in controls at the final sampling point. Importantly, the groups did not differ at baseline, and control calves did not show a significant change between the initial and final sample points. This difference appears to reflect a divergence in microbial trajectories during the study rather than a pre‐existing difference between groups. This finding is consistent with recent longitudinal studies demonstrating marked temporal changes in the relative abundance of Firmicutes and Bacteroidetes during early calf development, highlighting the dynamic nature of microbial assembly during this period (Robles‐Rodríguez et al. 2023; Gomes et al. 2024). LAB supplementation was associated with a significant divergence in Firmicutes abundance between treatment groups from initial to final sampling points, while Bacteroidetes showed a more temporally dynamic response. Their abundance increased significantly from the initial to final sampling point within LAB‐treated calves, while the initial‐to‐middle trajectory differed significantly between treatments, with higher Bacteroidetes abundance in LAB‐treated calves at the middle sampling point. This compositional trend is widely interpreted as indicative of a gut ecosystem increasingly optimized for carbohydrate fermentation and metabolic efficiency. In ruminants, Bacteroidetes are major members of the developing calf gut and include taxa that play a central role in the degradation of starch, fiber, and dietary protein, as well as in the downstream absorption of amino acids and peptides, thereby supporting short‐chain fatty acid (SCFA) production and energy harvest during the transition toward functional ruminancy (Malmuthuge et al. 2015; Meale et al. 2016). Firmicutes, by contrast, comprise a broad group of taxa capable of degrading complex carbohydrates that escape host digestion (Choudhary et al. 2021). While this functional capacity is essential within a balanced microbiome, disproportionate expansion of Firmicutes relative to Bacteroidetes has been associated with microbial imbalance and dysbiosis across species. In calves, elevated Firmicutes coupled with reduced Bacteroidetes abundance has been linked to diarrheal disease and impaired gut health (Fan et al. 2021), while similar shifts in humans have been associated with obesity, altered glucose metabolism, and gastrointestinal disorders (Schmidt et al. 2018; Magne et al. 2020).

A closer examination of genus‐level patterns provides additional insight into the specific microbial taxa through which LAB supplementation may have shaped the developing gut microbiota of calves. *Lactobacillus* showed a temporal pattern that is worth noting because of its direct relevance to the intervention. In control calves, *Lactobacillus* abundance decreased significantly from initial to final and from middle to final sampling points. In LAB‐treated calves, abundance decreased significantly from initial to middle, but there was no significant difference between initial and final sampling points. This pattern may be consistent with a degree of relative maintenance of *Lactobacillus*‐associated population during the latter phase in the treated calves compared with the decline observed in controls; similar temporal responses following probiotic supplementation have been reported previously (Ku et al. 2026). However, since no significant treatment‐by‐time interaction was detected in the present study, the difference in temporal patterns cannot be conclusively attributed to the LAB intervention. Nevertheless, it raises the possibility that supplementation may have influenced ecological conditions that supported resident *Lactobacillus* populations, a hypothesis that would require confirmation using strain‐resolved approaches such as strain‐specific quantitative PCR or shotgun metagenomic sequencing.

Post‐hoc within‐group comparisons also revealed several temporal changes in the control calves. The relative abundance of *Bacteroides, Parabacteroides, Mycoplasma, Shigella* and *Staphylococcus* decreased significantly between the middle and final sampling points, whereas *Helicobacter* increased significantly over the same period. Interestingly, these changes were not observed in the LAB‐treated calves. This contrasting pattern may suggest that LAB supplementation was associated with attenuation of some of the temporal fluctuations observed in the control microbiota. These observations are particularly relevant when considered alongside the significant phylum level treatment associated responses in Firmicutes and Bacteroidetes.

While the findings provide meaningful insight into the effects of LAB supplementation on the developing calf gut microbiota, some limitations should be acknowledged. Firstly, the probiotic intervention was administered over a relatively short duration of 30 days, which may have limited the ability to capture longer‐term microbial adaptation, persistence or functional maturation of the gut ecosystem. Consequently, the observed microbiota shifts should be interpreted as temporal responses during the intervention rather than definitive long‐term outcomes. Secondly, the number of calves available for inclusion in the study was constrained by herd size and farm management practices at the research facility during the study period. This limited sample size may have reduced statistical power to detect subtle treatment‐specific effects, particularly at the genus level. In addition, microbiota analyses were based on relative abundance profiling, which does not directly quantify absolute microbial abundance, microbial metabolic activity or functional outputs. As such, functional interpretations remain associative. Finally, although whole‐genome sequencing confirmed the absence of identified antimicrobial resistance and virulence determinants in the administered LAB strains and revealed bacteriocin‐associated genes, in vivo measurements of bacteriocin production and host physiological responses were not performed. Future studies incorporating larger cohorts, extended intervention periods, strain‐resolved sequencing, quantitative microbial measurement, metabolomic profiling, and host health outcomes will be important for validating the ecological patterns reported in this study.

In conclusion, this study demonstrates that supplementation with *Lactiplantibacillus pentosus* AA86 and *Pediococcus pentosaceus* AA94 was associated with selective temporal modulation of the early‐life gut microbiota of calves rather than a broad alteration of the microbial community. Although global diversity metrics remained unchanged, phylum‐level analyses identified significant treatment‐associated temporal responses involving Firmicutes and Bacteroidetes, while genus‐level revealed several contrasting temporal patterns between LAB‐treated and control calves despite a non‐significant treatment‐by‐time interactions. The relative preservation of *Lactobacillus* toward the final sampling point in LAB‐treated calves with the absence of several temporal shifts observed in the controls, suggests a possible influence of LAB supplementation on the temporal dynamics of the developing microbiota. However, these genus‐level observations remain explorative and may not be interpreted as definitive evidence of genus‐specific probiotic effect. Whole‐genome sequencing confirmed the absence of antimicrobial resistance and virulence determinants in the administered strains, while revealing the presence of bacteriocin‐encoding genes, providing a plausible microbiological basis for further investigation of their ability to interact with and modulate gut microbial communities. These findings are consistent with the possibility that early‐life probiotic interventions may influence the developing gut microbiota through ecosystem‐level interaction rather than through sustained changes in individual taxa. Overall, these findings suggest that early‐life LAB supplementation may influence selected components of calf gut microbiota development without substantially altering global microbial diversity or community structure. Future studies incorporating larger cohorts, longer follow‐up, strain‐resolved approaches, functional metagenomics and metabolomic analyses, and host health outcomes are needed to determine whether these taxonomic changes translate into measurable functional benefits and improved resilience to enteric disturbances during calf development.

## Author Contributions

**Adewale A. Adetoye:** investigation, writing – original draft, methodology, validation, visualization, writing – review and editing, data curation, formal analysis. **Ayorinde O. Afolayan:** writing – original draft, methodology, validation, visualization, writing – review and editing, formal analysis, data curation, software. **Isaac O. Olatoye:** methodology, validation, visualization, supervision, writing – review and editing. **Adedayo O. Faneye:** methodology, validation, visualization, writing – review and editing, supervision. **Funmilola A. Ayeni:** conceptualization, investigation, methodology, validation, visualization, writing – review and editing, formal analysis, project administration, supervision, resources.

## Funding

The authors have nothing to report.

## Ethics Statement

The authors have nothing to report.

## Conflicts of Interest

The authors declare no conflicts of interest.

## Data Availability

Whole‐genome sequencing data and 16S rRNA gene sequencing data generated in this study are publicly available in the National Center for Biotechnology Information Sequence Read Archive (SRA) under the BioProject accession numbers PRJNA1445101 and PRJNA1444915, respectively.
